# C‐Reactive Protein–Albumin–Lymphocyte (CALLY) Index as an Independent Risk Factor for Postoperative Atrial Fibrillation Recurrence

**DOI:** 10.1002/clc.70157

**Published:** 2025-06-02

**Authors:** Ye Deng, Jianya Huang, Li Deng, Yuxuan Zhou, Lu Pan, Jingyi Wang, Qianwen Chen, Qingqing Gu, Yang Zhang, Jun Wei, Ruxing Wang, Ling Sun, Yuan Ji, Qingjie Wang

**Affiliations:** ^1^ Department of Cardiology The Third Affiliated Hospital of Nanjing Medical University Changzhou Jiangsu China; ^2^ Department of Nephrology Fudan University Affiliated Shanghai Fifth People's Hospital Shanghai China; ^3^ Department of Cardiology The Affiliated Wuxi People's Hospital of Nanjing Medical University Wuxi Jiangsu China; ^4^ Department of Cardiovascular Surgery The Affiliated Hospital of Xuzhou Medical University Xuzhou China

**Keywords:** atrial fibrillation, C‐reactive protein, catheter ablation, inflammatory markers, recurrence

## Abstract

**Background:**

Atrial fibrillation (AF) recurrence after catheter ablation remains a clinical challenge despite guideline‐recommended efficacy. Emerging evidence implicates inflammatory biomarkers in predicting arrhythmia recurrence. This study investigated the novel CALLY index, a composite inflammatory marker, as a prognostic indicator for postablation AF recurrence.

**Methods:**

In this prospective cohort study, 556 consecutive AF patients undergoing catheter ablation (June 2018–June 2023) were stratified into recurrence and sinus rhythm (SR) maintenance groups. Cox regression and Kaplan–Meier analyses evaluated associations between the CALLY index and recurrence risk. Predictive accuracy was assessed via receiver operating characteristic (ROC) curves and area under the curve (AUC).

**Results:**

Over a median 12‐month follow‐up, 102 patients (18.3%) experienced recurrence. The SR group exhibited significantly higher CALLY indices than the recurrence group (3.24 ± 1.68 vs. 1.89 ± 0.57; *p* < 0.001). Univariate Cox regression identified the CALLY index as inversely associated with recurrence (HR: 0.439, 95% CI: 0.292–0.659; *p* < 0.001), with persistence after multivariable adjustment (HR: 0.887, 95% CI: 0.789–0.956; *p* = 0.031). Tertile‐based stratification revealed a 29% lower recurrence risk in the high‐CALLY group versus the low‐CALLY group (HR: 0.71, 95% CI: 0.68–0.76; *p* = 0.017). ROC analysis demonstrated optimal discrimination at a CALLY threshold ≥ 1.433 (AUC: 0.7899; sensitivity: 76.4%; specificity: 74.8%; *p* < 0.001).

**Conclusion:**

The CALLY index independently predicts AF recurrence postablation, offering potential utility in risk stratification. These findings support its integration into clinical decision‐making to optimize post‐procedural management.

## Graphical abstract

The CALLY index was identified as independent predictors of postablation recurrence in patients with atrial fibrillation. Our findings suggested that the CALLY index may serve as a novel prognostic marker for predicting arrhythmia recurrence following catheter ablation.

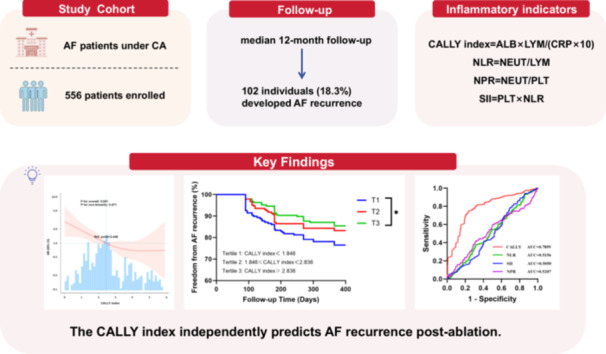

## 1 Introduction

Atrial fibrillation (AF), the predominant clinical arrhythmia worldwide, constitutes a major healthcare burden due to its strong association with thromboembolic events and cardiac dysfunction [1]. Epidemiological projections indicate China will face approximately 9 million AF cases among individuals ≥ 60 years by 2050, underscoring the urgency for effective management [2]. Although current guidelines endorse catheter ablation as first‐line therapy for drug‐refractory symptomatic AF (Class I recommendation), clinical outcomes remain suboptimal [3]. Post‐procedural recurrence rates reach 26.4% at 12‐month follow‐up, escalating to 30% in extended observation periods, highlighting critical limitations in current ablation strategies [4, 5].

Previous studies have demonstrated that inflammation plays a pivotal role in both the initiation and progression of AF [6]. Experimental models reveal that pro‐inflammatory cytokines facilitate structural reorganization of atrial tissue through fibroblast‐cardiomyocyte decoupling, thereby accelerating electrical and anatomical remodeling processes that predispose to postablation recurrence [7]. Contemporary clinical investigations further substantiate this relationship, demonstrating significant correlations between postprocedural arrhythmia recurrence and elevated circulating biomarkers, including high‐sensitivity CRP, neutrophil‐lymphocyte ratio (NLR), neutrophil‐platelet index (NPR), and systemic immune‐inflammation index (SII) [8, 9, 10, 11]. Furthermore, albumin is commonly considered a negative acute‐phase protein that tends to decrease during inflammatory conditions. A novel composite index, the CRP‐albumin‐lymphocyte (CALLY) index, has demonstrated superior predictive capability compared to conventional predictors for assessing the short‐ and long‐term major adverse cardiovascular events (MACEs) in patients with ST‐segment elevation myocardial infarction (STEMI) [12, 13]. Although the prognostic value of the CALLY index in STEMI has been established, the correlation between the pre‐ablation CALLY index and postablation recurrence in patients with AF remains unclear. Therefore, the current study aimed to systematically compare various compounding ratios of inflammatory markers to assess their association with postablation recurrence and further substantiate the utility of CALLY index as a predictor for postablation atrial fibrillation recurrence.

## 2 Methods

This was a single‐center, prospective, observational cohort study.

### 2.1 Ethical Considerations

The institutional review board at Affiliated Changzhou Second People's Hospital of Nanjing Medical University granted ethical approval (No.KY325‐01 and No.KY009‐01). Written informed consent was obtained from all participants preceding intervention. Research procedures adhered to ethical principles established in the Declaration of Helsinki (1964) and subsequent amendments.

### 2.2 Study Population

**Figure 1:**
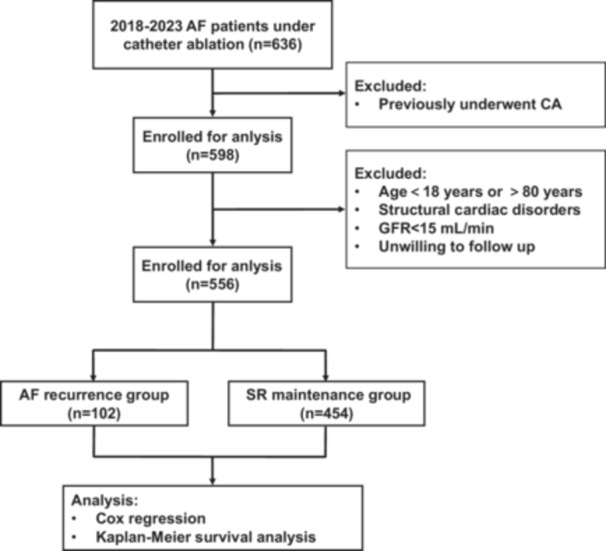
Study flow chart. AF, atrial fibrillation; CA, catheter ablation; GFR, glomerular filtration rate; SR, sinus rhythm.

This prospective cohort study included consecutive symptomatic AF patients receiving initial catheter ablation at the Third Affiliated Hospital of Nanjing Medical University from June 2018 to June 2023. Per contemporary classification standards, AF subtypes were categorized as paroxysmal (self‐terminating episodes ≤ 48 h) or persistent (sustained > 7 days, including pharmacologically/cardioversion‐terminated cases). Eligibility criteria comprised: (1) adults 18–80 years; (2) AF confirmation through 12‐lead ECG or 24‐h Holter monitoring; (3) documented persistent/paroxysmal AF subtypes; (4) absence of class I/III antiarrhythmic medication use. Exclusion criteria involved: (1) prior ablation procedures; (2) structural cardiac abnormalities; (3) end‐stage renal failure requiring dialysis; (4) non‐adherence to follow‐up protocols (see Figure 1 for screening flowchart).

### 2.3 Laboratory Testing and Echocardiography

Pre‐ablation blood testing was performed following 12‐h fasting protocols, with antecubital venipuncture specimens collected in K3EDTA anticoagulated tubes (Becton Dickinson) during morning hours (07:00–09:00 a.m.). Complete blood count analysis determined leukocyte differentials (neutrophil‐lymphocyte ratio), platelet levels, and hematological indices. Comprehensive metabolic profiling evaluated: (1) lipid panel (TG, TCH, LDL‐C); (2) cardiac enzymes (CK‐MB); (3) inflammatory markers (CRP); (4) hepatic/renal function biomarkers; (5) coagulation parameters; (6) thyroid function tests. Preprocedural echocardiographic evaluation quantified cardiac dimensions: aortic annulus diameter (AO), diastolic LV internal diameter (LVIDd), interventricular septal thickness (IVSd), posterior wall dimension (LVPWd), systolic LV diameter (LVIDs), left atrial size (LAD), along with functional assessments of LVEF and FS using Simpson's biplane method.

The CALLY index was calculated using the following formula: CALLYindex=albumin×lymphocytecount/(CRP×10). Other complex inflammatory indicators were calculated as follows: NLR = neutrophil count/lymphocyte count, NPR = neutrophil count/platelet count, SII = platelet count × NLR [12].

### 2.4 Catheter Ablation of Atrial Fibrillation

Per protocol, direct oral anticoagulants (DOACs) were prescribed for ≥ 3 weeks preprocedural anticoagulation [14]. Preprocedural transesophageal echocardiography (TEE) systematically excluded left atrial thrombi [15, 16, 17, 18]. Antiarrhythmic medications were discontinued for ≥ 5 elimination half‐lives before intervention [19].

The ablation protocol commenced with bilateral femoral venous access. Two 8.5‐F steerable introducers (SL1, Abbott Laboratories) were positioned in the left atrial chamber following fluoroscopy‐guided transseptal puncture. Systemic anticoagulation was initiated with intravenous heparin titrated to achieve activated clotting times (ACT) of 300–350 s [20, 21]. Three‐dimensional electroanatomic mapping (CARTO3, Biosense Webster) was performed using a high‐density mapping catheter (Pentaray, Biosense Webster) to reconstruct atrial geometry. All interventions were performed by three board‐certified electrophysiologists (individual experience > 300 AF ablations) with extensive expertise in contact‐force sensing catheter applications (SmartTouch usage > 20 cases per operator).

The ablation protocol employed a systematic workflow utilizing AI‐optimized parameters. Circumferential pulmonary vein isolation was executed with the SmartTouch ablation catheter (Biosense Webster, CA, USA), incorporating real‐time contact force monitoring (5–30 g) and power titration (30–45 W). Radiofrequency lesions were sequentially deployed with 4‐mm intertag spacing to ensure circumferential continuity. Anatomically stratified AI targets were implemented: anterior wall 500, roof 400–450, posterior/inferior regions 350–400. Sustained arrhythmia post‐isolation mandated synchronized direct‐current cardioversion (200 J biphasic). Subsequent substrate modification was conducted in cases demonstrating left atrial low‐voltage zones (< 0.5 mV) identified through high‐density mapping.

### 2.5 Follow‐Up and Outcome

Postablation management protocol mandated a 90‐day course of rhythm‐control medications (absent contraindications) with scheduled outpatient surveillance [22]. Systematic follow‐up evaluations comprised clinical assessments and 24‐h ambulatory ECG monitoring at quarterly intervals (3, 6, 9, 12 months). Symptom‐triggered 12‐lead ECGs were immediately acquired for patients reporting palpitations or syncope. AF recurrence was defined as documented episodes of AF lasting > 30 s following a 3‐month blanking period [23].

### 2.6 Statistical Analysis

Data analysis was conducted using IBM SPSS Statistics (v27.0, IBM, NY). Quantitative parameters conforming to normal distribution were presented as mean ± standard deviation, while skewed data were expressed as median (interquartile range). Categorical data were summarized as frequencies (percentages). Group comparisons employed Student's *t‐*test for normally distributed variables and Pearson's *χ* ^2^ test for proportions. Univariate screening of predictors preceded multivariable Cox proportional hazards modeling, retaining variables with *p* < 0.05 in initial analyses. Survival probabilities were estimated through Kaplan–Meier methodology with CALLY index tertile stratification, compared via Mantel–Cox log‐rank testing. Three sequential Cox models were constructed: Model 1 (unadjusted), Model 2 (adjusted for demographic covariates), and Model 3 (full adjustment including biochemical/imaging parameters). Diagnostic accuracy of inflammatory indices was assessed through ROC curve analysis (AUC comparison). A Comparison of predictive performance between ROC curves was conducted using DeLong's non‐parametric test for paired receiver operating characteristic curves, implemented through the roc.test function in the R statistical environment (version 4.2.1) with the pROC package (version 1.18.0). All inferences used two‐tailed tests with *α* = 0.05 and *p* < 0.05 was considered to be statistically significant.

## 3 Results

Following the exclusion of ineligible candidates, this prospective cohort study enrolled 556 treatment‐naive AF patients (326 males, 230 females) undergoing initial catheter ablation. All participants completed a minimum 12‐month follow‐up, during which 102 individuals (18.3%) developed recurrent AF. The cohort was stratified into two groups based on rhythm outcomes: the AF recurrence group (*n* = 102) and the SR maintenance group (*n* = 454).

As detailed in Table 1, significant intergroup disparities emerged across multiple clinical indices. The AF recurrence cohort exhibited elevated body mass index (BMI), C‐reactive protein (CRP), triglyceride (TG), and thyroid‐stimulating hormone (TSH) levels, alongside greater left atrial dimensions (all *p* < 0.05). Conversely, this group demonstrated younger age, reduced lymphocyte counts, lower thrombin time (TT), and diminished total protein (TP) concentrations compared to the SR maintenance group (all *p* < 0.05). A lower prevalence of coronary artery disease (CAD) was observed in the AF recurrence cohort (*p* < 0.05). No significant differences existed between groups regarding sex distribution, AF subtype classification, or left ventricular ejection fraction.

**Table 1:** Baseline characteristics of enrolled patients.

	AF recurrence group (*n* = 102)	SR maintenance group (*n* = 454)	*p* value
Demographics			
Male, *n* (%)	55 (53.92)	271 (59.69)	0.285
Age (year)	65.22 ± 9.629	65.07 ± 9.469	0.006
BMI (kg/m²)	25.84 ± 2.88	24.79 ± 3.04	0.002
Persistent AF, *n* (%)	35 (34.31)	176 (38.77)	0.402
Comorbidities			
Alcohol consumption, *n* (%)	15 (14.71)	55 (12.11)	0.476
Current smoking, *n* (%)	29 (28.43)	113 (24.89)	0.466
Hypertension, *n* (%)	62 (60.78)	308 (67.84)	0.172
CAD, *n* (%)	6 (5.88)	96 (21.15)	< 0.001
Diabetes, *n* (%)	25 (24.51)	95 (20.91)	0.426
Hypothyroidism, *n* (%)	3 (2.94)	14 (3.10)	0.94
Laboratory values			
WBC (×10^9/L)	6.43 ± 1.98	6.44 ± 1.79	0.915
NEUT%	78.77 ± 13.36	61.42 ± 10.25	0.63
L (×10^9/L)	1.95 ± 0.45	2.20 ± 0.41	< 0.001
RBC (×10^9/L)	4.51 ± 0.51	5.43 ± 10.64	0.385
Hb (g/L)	138.96 ± 16.01	140.05 ± 19.25	0.595
PLT (×10^9/L)	196.95 ± 57.89	196.26 ± 52.47	0.906
RDW‐CV (%)	13.26 ± 2.78	13.01 ± 1.82	0.274
CRP (mg/L)	12.12 ± 4.36	4.04 ± 1.47	< 0.001
PT (s)	14.73 ± 6.19	14.81 ± 7.15	0.959
INR	1.30 ± 0.54	1.28 ± 1.23	0.911
APTT (s)	32.00 ± 7.67	31.14 ± 8.59	0.350
TT (s)	22.07 ± 3.97	25.46 ± 4.84	0.031
FBG (mg/dL)	105.27 ± 32.32	103.48 ± 31.21	0.605
Total protein (g/L)	72.45 ± 4.42	77.43 ± 4.24	< 0.001
ALB (g/L)	45.83 ± 3.73	47.52 ± 4.08	0.258
GLB (g/L)	26.61 ± 4.22	29.90 ± 5.06	0.367
CPK (U/L)	83.47 ± 4.38	86.15 ± 5.17	0.615
CK‐MB (U/L)	15.22 ± 6.97	15.34 ± 6.04	0.928
BUN (mmol/L)	5.68 ± 1.45	7.26 ± 1.69	0.422
Cr (μmol/L)	75.45 ± 17.33	76.57 ± 44.79	0.803
UA (μmol/L)	322.29 ± 79.40	333.28 ± 90.21	0.257
TCH (mmol/L)	4.20 ± 1.00	4.11 ± 0.98	0.368
TG (mmol/L)	2.75 ± 1.67	1.56 ± 1.10	0.020
LDL‐C (mmol/L)	2.33 ± 0.80	2.30 ± 0.76	0.755
TSH (μIU/L)	2.84 ± 3.30	2.32 ± 2.02	0.039
FT3 (pmol/L)	4.91 ± 2.26	6.90 ± 31.20	0.521
FT4 (pmol/L)	16.49 ± 4.39	17.21 ± 3.67	0.084
BNP (pg/mL)	25.84 ± 2.91	24.79 ± 3.07	0.15
Echocardiography			
IVSd (mm)	9.64 ± 1.46	9.78 ± 4.20	0.734
LVIDd (mm)	50.08 ± 4.01	50.43 ± 4.54	0.47
LVPWD (mm)	9.35 ± 0.93	9.35 ± 1.02	0.832
LVIDs (mm)	34.56 ± 3.64	35.42 ± 14.78	0.559
AO (mm)	30.76 ± 2.66	31.32 ± 3.13	0.097
LAd (mm)	44.47 ± 6.07	42.44 ± 5.38	0.047
FS (%)	31.16 ± 3.71	31.41 ± 4.43	0.592
LVEF (%)	58.46 ± 5.67	58.61 ± 6.13	0.825
Inflammatory indicators			
NPR	0.32 ± 0.05	0.31 ± 0.04	0.511
NLR	4.06 ± 1.81	5.34 ± 2.78	0.642
SII	816.33 ± 468.89	1074.70 ± 557.32	0.64
CALLY index	1.89 ± 0.57	3.24 ± 1.68	< 0.001

*Note:* Data are presented as the mean ± standard deviation or median (interquartile range) and counts (percentages).

Abbreviations: AF, atrial fibrillation; ALB, albumin; AO, aorta; APTT, activated partial thromboplastin time; BMI, body mass index; BNP, Brain natriuretic peptide; BUN, blood urea nitrogen; CAD, coronary artery disease; CPK, creatine phosphokinase; Cr, creatinine; CRP, C‐reactive protein; FBG, fasting blood glucose; FS, Fractional shortening; FT3, free triiodothyronine; FT4, free thyroxine; GLB, globulin; Hb, hemoglobin; IVSd, Interventricular septum thickness in diastole; L, lymphocyte; LDL‐C, low‐density lipoprotein cholesterol; LVIDd, Left ventricular internal diameter in diastole; LVPWD, Left ventricular posterior wall thickness in diastole; LVIDs, Left ventricular internal diameter in systole; LAd, left atrial diameter; LVEF, left ventricular ejection fraction; NEUT, neutrophil count; PLT, blood platelet; PT, prothrombin time; RBC, red blood cell; SR, sinus rhythm; TCH, total cholesterol; TG, triglyceride; TSH, thyroid‐stimulating hormone; TT, thrombin time; UA, uric acid; WBC, white blood cell.

**Figure 2:**
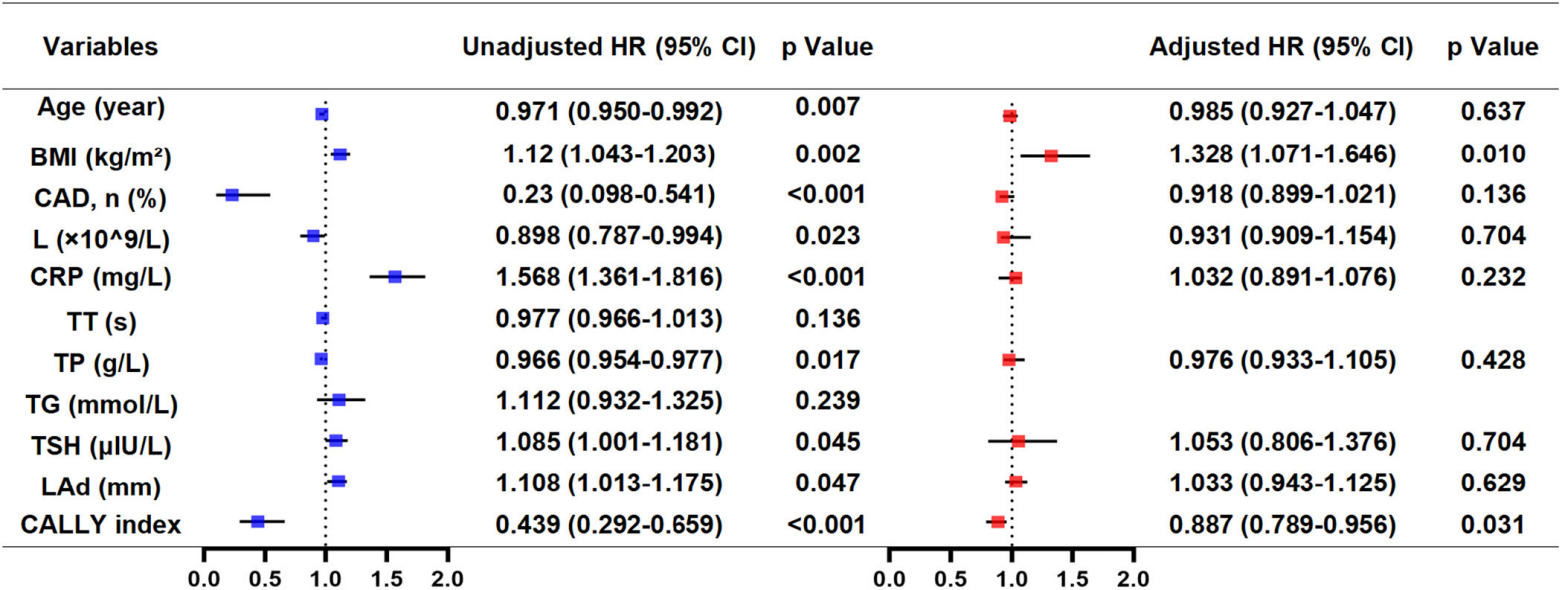
Univariate and multivariate COX regression analyses of multiple variables on recurrence after catheter ablation.

Univariate Cox regression identified nine parameters significantly associated with postablation AF recurrence (Figure 2). Protective factors included advancing age (HR: 0.971, 95% CI: 0.950–0.992), CAD history (HR: 0.230, 95% CI: 0.098–0.541), elevated TP levels (HR: 0.966, 95% CI: 0.954–0.977) and CALLY index (HR: 0.439, 95% CI: 0.292–0.659). Conversely, BMI (HR: 1.120, 95% CI: 1.043–1.203), lymphocyte counts (HR: 1.118, 95% CI: 1.087–1.194), CRP (HR: 1.568, 95% CI: 1.361–1.816), TSH (HR: 1.085, 95% CI: 1.001–1.181), left atrial diameter (HR: 1.1088, 95% CI: 1.013–1.175) emerged as risk predictors (all *p* < 0.05). Multivariable analysis incorporating these variables revealed BMI (adjusted HR: 1.328, 95% CI: 1.071–1.646, *p* = 0.010) and CALLY index (adjusted HR: 0.887, 95% CI: 0.789–0.956, *p* = 0.031) as independent prognostic indicators.

**Figure 3:**
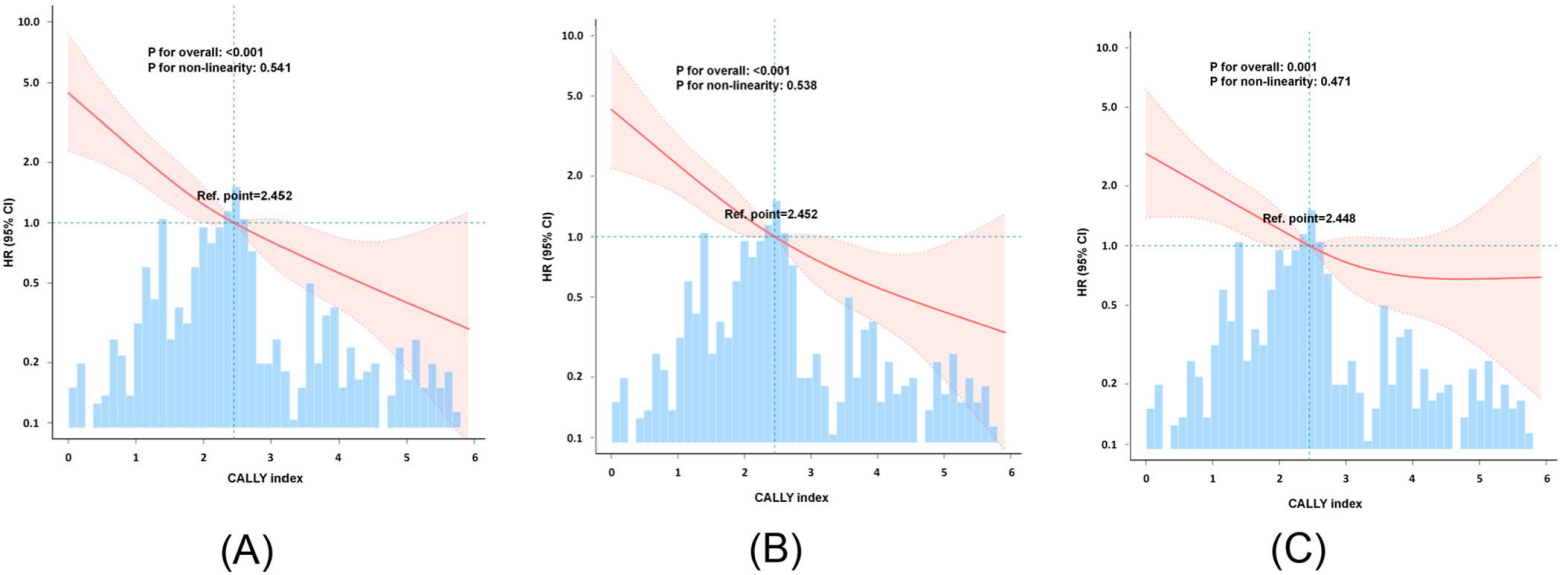
The relation of the CALLY index with the risk of AF recurrence in a restricted cubic splines model. (A) is not adjusted, (B) is adjusted for age, sex, and BMI, and (C) is adjusted for age, sex, BMI, TG, TP, TSH, CAD history, left atrial diameter, and CRP.

To explore the potential nonlinear relationship between the CALLY index and AF recurrence, we fitted restricted cubic splines (RCS). Figure 3A presents the unadjusted model, Figure 3B is adjusted for age, sex, and BMI, and Figure 3C is further adjusted for age, sex, BMI, TG, TP, TSH, CAD history, left atrial diameter, and CRP. The RCS analysis indicated an approximately linear relationship between the CALLY index and AF recurrence (*p* > 0.05).

**Figure 4:**
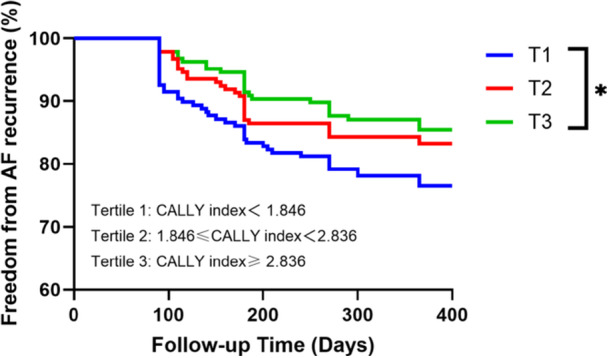
Kaplan–Meier curves for AF recurrence across tertiles of CALLY index. It was divided into three groups, tertile 1 defined as the low CALLY group (CALLY index＜1.846), tertile 2 as the moderate CALLY group (1.846 ≤ CALLY index＜2.836), and tertile 3 as the high CALLY group (CALLY index≥ 2.836). Statistical significance denoted by *p < 0.05. AF, atrial fibrillation.

Participants were categorized into CALLY index tertiles: Tertile 1 (lowest, reference), Tertile 2 (moderate), and Tertile 3 (highest). Unadjusted Cox models demonstrated progressive risk reduction across tertiles (Tertile 2: HR: 0.51, 95% CI: 0.47–0.78; Tertile 3: HR: 0.60, 95% CI: 0.52–0.73; both *p* < 0.001). These associations persisted after sequential adjustment for: (1) Demographic factors (age, sex, BMI); (2) Comprehensive covariates (age, sex, BMI, TG, TP, TSH, CAD history, left atrial diameter, CRP). Final adjusted models confirmed sustained protective effects in Tertile 3 (HR: 0.71, 95% CI: 0.68–0.76, *p* = 0.017) relative to Tertile 1 (Table 2). Kaplan–Meier survival curves corroborated these findings, revealing significantly higher recurrence rates in the low CALLY group (log‐rank *χ*² = 4.701, *p* = 0.03; Figure 4). Subgroup analysis by AF subtype revealed that the CALLY index's predictive performance was consistent between paroxysmal (HR: 0.892, 95% CI: 0.785–0.964, *p* = 0.029) and persistent (HR: 0.879, 95% CI: 0.771–0.951, *p* = 0.034) AF, with no significant interaction (*p* = 0.67; Supporting Information S1: Table S1).

**Table 2:** Hazard ratios for recurrent AF after catheter ablation according to CALLY index Tertiles.

Variables		Tertile		
		T1	T2	T3
Median and tertile value		< 1.846	1.846–2.836	≥ 2.836
Model 1	Hazard ratio	1	0.51	0.60
	95% CI	—	0.47–0.78	0.52–0.73
	*p* value	—	< 0.001	< 0.001
Model 2	Hazard ratio	1	0.47	0.39
	95% CI	—	0.36–0.78	0.32–0.69
	*p* value	—	< 0.001	< 0.001
Model 3	Hazard ratio	1	0.84	0.71
	95% CI	—	0.59–1.11	0.68–0.76
	*p* value	—	0.128	0.017

*Note:* Model 1 was not adjusted, Model 2 adjusted for age, sex, and BMI, and Model 3 adjusted for age, sex, BMI, TG, TSH, CAD, TP, LAd, and CRP. According to the CALLY Tertile, it was divided into three groups, Tertile 1 defined as the low CALLY group (CALLY index < 1.8460), Tertile 2 as the moderate CALLY group (1.846 ≤ CALLY index < 2.836), and Tertile 3 as the high CALLY group (CALLY index ≥ 2.836).

**Figure 5:**
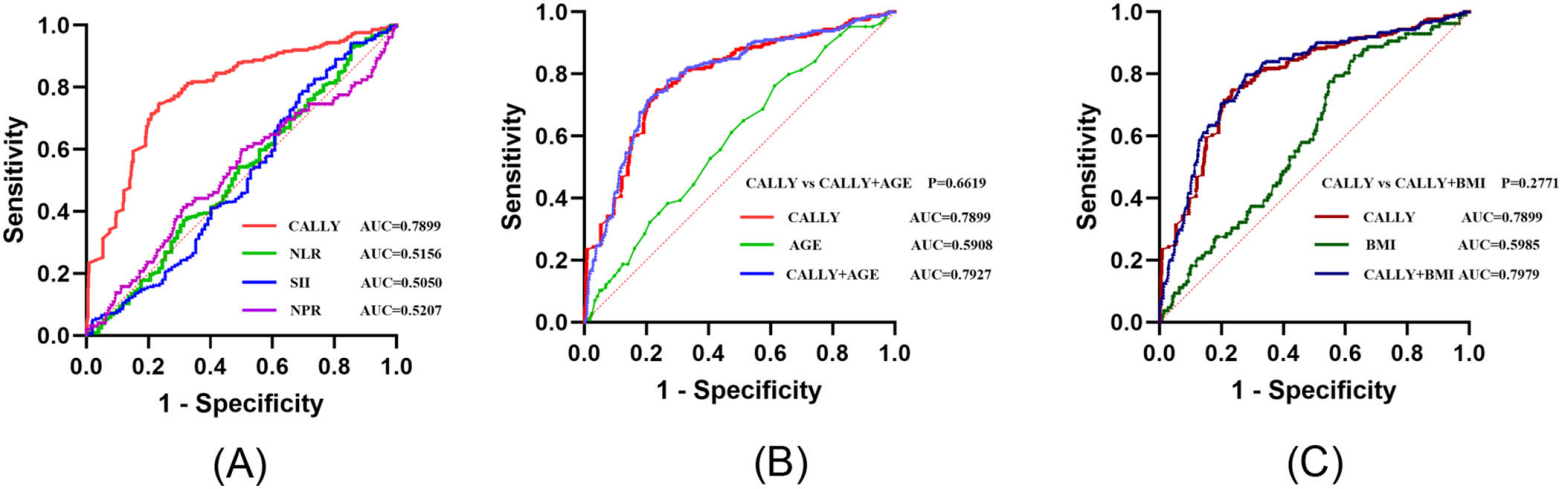
Receiver operating characteristic (ROC) curves for CALLY (c‐reactive protein‐albumin‐lymphocyte) index, NLR (neutrophil to lymphocyte ratio), NPR (neutrophil to platelet ratio), SII (systemic immune‐inflammation index), age, and BMI in predicting recurrence of AF. (A) The highest area under the curve (AUC) value was observed in ROC curve of CALLY index (AUC: 0.7899, *p* < 0.001). (B) The combination of CALLY index and age showed an AUC of 0.7927, while the efficiency in predicting recurrence of AF is the same by CALLY index alone (*p* = 0.6619). (C) The combination of CALLY index and BMI showed an AUC of 0.7979, while the efficiency in predicting recurrence of AF is the same by CALLY index alone (*p* = 0.2771). Abbreviation: AF, atrial fibrillation.

Receiver operating characteristic (ROC) analysis evaluated four inflammatory indices for AF recurrence prediction (Figure 5A). The CALLY index demonstrated superior discriminative capacity (AUC: 0.7899, *p* < 0.001), outperforming NLR, NPR, and SII. Optimal CALLY cutoff ≥ 1.433 provided 76.4% sensitivity and 74.8% specificity for identifying recurrence risk. The incorporation of age or BMI into the CALLY index improved AUC but did not yield a statistically significant improvement in predictive performance (*p* > 0.05), as shown in Figure 5B,C.

## 4 Discussion

In this study, the prognostic value of the CALLY index in predicting postablation recurrence in patients with AF was evaluated. The key findings of this study are discussed below. First, after adjusting for confounding factors through multivariate regression analysis, the elevated CALLY index emerged as an independent protective factor against the recurrence of AF patients, and elevated BMI as an independent risk factor. And, the ROC curve analysis demonstrated that the CALLY index exhibited favorable predictive accuracy for postablation recurrence in AF patients.

The result of this study indicated a significant association between elevated BMI and the recurrence of AF. This finding is consistent with previous reports, suggesting that obesity may promote both the onset and persistence of AF through multiple mechanisms [24, 25]. First, obesity is often accompanied by structural cardiac alterations, such as left atrial enlargement and fibrosis, which provide a structural substrate for AF development [26, 27, 28]. Furthermore, excessive fat accumulation, particularly an increase in epicardial adipose tissue, can induce local inflammatory responses, and the resultant secretion of pro‐inflammatory cytokines may further trigger electrophysiological abnormalities in the myocardium, thereby elevating the risk of AF recurrence [29, 30, 31]. In addition, obesity is frequently linked with metabolic disturbances (such as insulin resistance and hyperlipidemia) as well as comorbid conditions, including hypertension and diabetes. These factors may interact synergistically to exacerbate both electrophysiological and structural remodeling of the atria [32, 33, 34].

Emerging evidence underscores the pathophysiological interplay between inflammatory cascades and electrical substrate modification in atrial cardiomyopathy. Compelling experimental data reveal that pro‐inflammatory cytokines can generate ectopic focal discharges with electrophysiological potency comparable to pulmonary vein triggers [35, 36]. While the association between systemic inflammation and de novo AF pathogenesis is well‐characterized, the paucity of evidence persists regarding postablation inflammatory predictors. A seminal meta‐analysis demonstrated quantitative CRP elevation proportional to AF duration, with permanent AF patients exhibiting 42% higher CRP than paroxysmal cases (95% CI 1.25–1.61), both cohorts surpassing non‐AF controls [37]. Our findings corroborate this inflammatory continuum, revealing quantitatively superior CRP concentrations in recurrence cases, potentially reflecting both elevated preprocedural AF burden and persistent inflammatory milieu conducive to arrhythmogenesis. The mechanistic link between CRP and AF recurrence may involve multiple pathways. CRP has been shown to directly promote atrial structural remodeling through fibroblast activation and extracellular matrix deposition, creating a substrate conducive to reentrant arrhythmias [38]. CRP may exacerbate oxidative stress and endothelial dysfunction, thereby altering atrial electrophysiological properties and facilitating triggered activity [39, 40]. Furthermore, elevated CRP levels could reflect underlying comorbidities that perpetuate systemic inflammation and atrial myopathy.

As a vital component of the immune regulatory system, abnormalities in lymphocyte quantity and function can directly or indirectly influence the inflammatory state and structural remodeling of the atria [41]. Under certain conditions, the secretion of various cytokines may promote atrial fibrosis and electrophysiological abnormalities, thereby increasing the risk of AF recurrence. Moreover, some studies have reported an imbalance in specific lymphocyte subsets, such as CD4+ and CD8+ T cells, in patients with AF, and this immune dysregulation may weaken the suppression of local inflammation, leading to a chronic inflammatory state in atrial tissue. Our analysis identified a statistically significant reduction in circulating lymphocyte concentrations within the AF recurrence group compared to the SR maintenance group. This immunological disparity persisted after multivariable adjustment for confounding hematological parameters. Reduced lymphocyte levels may indicate impaired immune function or a local inflammatory imbalance, potentially leading to alterations in atrial structure and electrophysiological properties, thereby exacerbating atrial remodeling and increasing the risk of AF recurrence.

As a hepatically synthesized negative acute‐phase protein, albumin demonstrates inverse correlations with systemic inflammation intensity [42]. Epidemiological evidence reveals progressive cardiovascular risk escalation with declining albumin levels, particularly manifesting as a 23% increased AF incidence per 5 g/L decrement (95% CI 1.12–1.35) across cardiometabolic disease cohorts. Beyond its role as an inflammation biomarker, albumin modulates multiple hemodynamic parameters through viscosity regulation, endothelial nitric oxide bioavailability enhancement, and platelet aggregation inhibition [43, 44]. As the CALLY index integrates three key inflammatory biomarkers, it may offer greater specificity in patients with inflammatory disorders. Previous validation in acute coronary syndromes confirmed its STEMI prognostic utility [12]. Our findings revealed comparable albumin concentrations between AF recurrence and SR maintenance groups, indicating that the observed CALLY index differential primarily stems from combined CRP elevation and lymphocytosis in recurrence cases.

## 5 Limitations

This study has several limitations that should be acknowledged. First, blood parameters were collected only at baseline during initial hospitalization, with no subsequent measurements during postoperative follow‐up. This precluded the assessment of dynamic changes in inflammatory markers, such as the CALLY index, postablation. Second, the lack of comprehensive baseline phenotyping for key comorbidities, such as sleep‐disordered breathing, may introduce residual confounding. Third, the 12‐month follow‐up period may not fully capture the natural history of postablation atrial fibrillation (AF) recurrence, as delayed electrical remodeling may manifest beyond this timeframe. Fourth, the single‐center design, conducted in a Chinese population, may limit the generalizability of the CALLY index's predictive performance to diverse populations with differing AF prevalence, comorbidity profiles (e.g., higher obesity rates in Western cohorts), or ablation protocols. To address these limitations, future multicenter studies with extended follow‐up, serial biomarker assessments, and comprehensive comorbidity screening are warranted to validate the CALLY index's prognostic utility across varied clinical settings.

## 6 Conclusion

The CALLY index was identified as independent predictors of postablation recurrence in patients with atrial fibrillation. Notably, these findings suggest that the CALLY index may serve as a novel prognostic marker for predicting arrhythmia recurrence following catheter ablation. However, further validation through large‐scale, prospective clinical trials is essential to confirm the clinical utility and prognostic significance of these metrics.

## Consent

The authors have nothing to report.

## Conflicts of Interest

The authors declare no conflicts of interest.

## Supporting information

Supplementary Material‐dy‐0508.

## Data Availability

Data supporting the findings of this study are available from the corresponding author upon reasonable request.
