# Supplementary material for: C‐Reactive Protein–Albumin–Lymphocyte (CALLY) Index as an Independent Risk Factor for Postoperative Atrial Fibrillation Recurrence
Source: Clin Cardiol. 2025 Jun 2;48(6):e70157. doi: 10.1002/clc.70157 (PMC12128145; doi:10.1002/clc.70157)
Supplement: Supplementary file 1 — Supplementary Material‐dy‐0508. [file CLC-48-e70157-s001.docx]

**TABLE S1. Subgroup analysis of CALLY Index’ s predictive performance for AF recurrence by AF subtype**

| AF subtype | N | HR (95% CI) | P Value | p for interaction |
| --- | --- | --- | --- | --- |
| Paroxysmal | 345 | 0.892 (0.785–0.964) | 0.029 | 0.67 |
| Persistent | 211 | 0.879 (0.771–0.951) | 0.034 |  |

Note: Hazard ratios (HR) and 95% confidence intervals (CI) were derived from multivariable Cox regression models adjusted for age, sex, BMI, TG, TP, TSH, CAD history, left atrial diameter, and CRP. The p-value for interaction tests whether the CALLY index’s predictive performance differs significantly between paroxysmal and persistent AF subtypes. AF, atrial fibrillation.
